# 4436 A Content Analysis of CTSA Websites: The Identification and Evaluation of CTSA Program Hub Website Content Standards for Knowledge Management of NCATS CTSA Program Goals and Initiatives

**DOI:** 10.1017/cts.2020.351

**Published:** 2020-07-29

**Authors:** Barbara Ann Tafuto, Reynold Panettieri, James Scott Parrot, Shankar Srinivasan, Kristi Holmes, Dagobert Soergel

**Affiliations:** 1 Rutgers University; 2 Northwestern University; 3 University of Buffalo

## Abstract

OBJECTIVES/GOALS: Introduction: Between 2014 and 2019 the National Institute of Health (NIH) through the National Center for the Advancement of Translational Science (NCATS) has awarded about $2.7 billion to U.S. Academic Medical Centers to build a national network of clinical and translational science program hubs that serve to meet their key goals and initiatives. Today there are about 60 Clinical and Translational Science Award (CTSA) program hubs. Each CTSA program hub has a corresponding website highlighting its clinical and translational science centered programs and activities. These websites are a critical communication gateway to promote NCATS goals and initiatives. Objective: The objective of this research is to evaluate the NIH funded Clinical and Translational Science Award (CTSA) program hub websites for NCATS goals and initiative content alignment, navigability, and interactivity. METHODS/STUDY POPULATION: Methods: Each CTSA program hub website was systematically evaluated for information or tools that align with the five NCATS / CTSA Goals and eight CTSA nationally identified program initiatives. Each NCATS goal and CTSA initiative was subsequently ranked by information diversity level (text, tool, interactivity) and navigation level (click distance from the home page). RESULTS/ANTICIPATED RESULTS: Results: Four of the five NCATS goals are thoroughly and consistently represented among the CTSA Consortium with workforce development, patient and community engagement, and quality and efficiency of research being the top three. Informatics is thoroughly and consistently represented, but not always clearly identified on the home page. The most underrepresented goal is integration of special and underserved populations which was identified on only 60% of CTSA program hub websites. The most common focus of the eight CTSA program initiatives is the Trial Innovation Network in CTSA program hub websites. The Smart IRB comes in a distant second. The remaining six initiatives are severely underrepresented. DISCUSSION/SIGNIFICANCE OF IMPACT: Discussion: The identification of these gaps among the CTSA program hubs presents an understanding of content management and website functionality among the consortium from 3 principal approaches. First it creates an understanding of CTSA program hub content alignment with its funding source goals and initiatives. Such an understanding presents an opportunity to promote ways to create a better aligned consortium with improved collaboration pathways by the funding source through program hub website content standards. Second, it creates an opportunity for program hubs to understand and respond to the messaging their websites are presenting as it relates to the funding source. Third, it provides an opportunity to identify specific program initiatives and goals the CTSA institutions independently chose to highlight which can open a dialog to the better understanding the value of the program initiatives as they relate to the needs of CTSA program hubs. Ultimately, CTSA websites through content alignment should lead to an improved user experience.

